# Insulin Growth Factor Binding Protein-6 and the Liver

**DOI:** 10.3390/cells15010077

**Published:** 2026-01-02

**Authors:** Anna Rita Daniela Coda, Sławomir Kasperczyk, Michał Dobrakowski, Aleksandra Kasperczyk, Maria Incoronata Trecca, Arcangelo Liso, Gaetano Serviddio, Francesco Bellanti

**Affiliations:** 1 C.R.E.A.T.E.—Center for Research and Innovation in Medicine, Department of Medical and Surgical Sciences, University of Foggia, 71122 Foggia, Italy; daniela.coda@unifg.it (A.R.D.C.); incoronata.trecca@unifg.it (M.I.T.); gaetano.serviddio@unifg.it (G.S.); 2 Department of Biochemistry, Faculty of Medical Sciences in Zabrze, Medical University of Silesia, 41-808 Katowice, Poland; skasperczyk@sum.edu.pl (S.K.); olakasp@poczta.onet.pl (A.K.); 3 Department of Radiology and Radiodiagnostics, Faculty of Medical Sciences in Zabrze, Medical University of Silesia, 41-808 Katowice, Poland; michal.dobrakowski@poczta.fm; 4 Department of Medicine and Surgery, University of Perugia, 06123 Perugia, Italy; arcangelo.liso@unipg.it

**Keywords:** IGFBP-6, IGF-II, IGF-1R signaling, hepatic stellate cells, lobular zonation, liver fibrosis, hepatocellular carcinoma, cholangiocarcinoma, post-translational modification, spatial transcriptomics

## Abstract

**Highlights:**

**What are the main findings?**

- IGFBP-6 is a context-dependent regulator in the liver—enriched in stellate/cancer-associated fibroblast (CAF) compartments and shaped by lobular zonation—controlling IGF-II availability and exerting IGF-independent actions.
- Profibrotic, inflammatory, hypoxia/redox, and metabolic cues (including PTMs like O-GlcNAc) dynamically tune IGFBP-6 abundance and function across disease states and liver cancers.

**What are the implications of the main findings?**

- Research and clinical assays should shift from bulk abundance to function + location—PTM-aware proteomics, extracellular-vesicle profiling, and spatial readouts—to correctly interpret IGFBP-6 and IGF-II activity.
- Translational strategies include composite biomarkers and patient selection frameworks that use total/modified IGFBP-6 to guide IGF-axis therapies or microenvironment-targeted approaches.

**Abstract:**

The insulin-like growth factor (IGF) axis orchestrates hepatic development, regeneration, and metabolism, yet the roles of individual IGF-binding proteins (IGFBPs) remain incompletely defined. IGFBP-6, a high-affinity, IGF-II-preferring binding protein, has emerged as a context-dependent modulator of IGF bioavailability and cell signaling with additional IGF-independent actions. This review synthesizes current evidence on IGFBP-6 in liver biology and disease. We first outline hepatic expression, regulation, and post-translational processing of IGFBP-6 across development, homeostasis, and injury, and summarize its effects on canonical IGF-II/IGF1R signaling and downstream phosphatidylinositol 3-kinase—protein kinase B (PI3K–AKT) and rat sarcoma—mitogen-activated protein kinase (RAS–MAPK) pathways. We then evaluate experimental and clinical data linking IGFBP-6 to steatotic liver disease, inflammation, and fibrogenesis, including putative roles in hepatocyte stress responses, stellate cell activation, and extracellular matrix remodeling. Finally, we examine IGFBP-6 in primary liver cancers—hepatocellular carcinoma and cholangiocarcinoma—highlighting evidence for tumor-suppressive versus pro-migratory activities, potential crosstalk with hypoxia, Wnt/β-catenin and TGF-β signaling, and interactions with the tumor immune microenvironment. Across conditions, we assess the translational potential of IGFBP-6 as a circulating or tissue biomarker, its utility for patient stratification, and prospects for therapeutic targeting—either by modulating IGF-II sequestration or exploiting IGF-independent mechanisms. We conclude by identifying key knowledge gaps, methodological limitations, and priorities for future studies, including standardized measurement, cell-type-resolved profiling, and in vivo perturbation in clinically relevant models. Collectively, the review positions IGFBP-6 as a nuanced regulator of liver pathophysiology and a promising, yet underexplored, lever for diagnosis and therapy.

## 1. Introduction

Insulin-like growth factor (IGF) signaling is embedded in endocrine and paracrine circuits that shape tissue growth and repair; within this system, binding proteins gate ligand availability and diversify biological outcomes beyond receptor activation alone [1,2]. Although the oncologic literature has emphasized receptor-centric strategies, accumulating evidence points to context-specific regulation by IGF-binding proteins (IGFBPs)—including ligand buffering, extracellular-matrix interactions, and IGF-independent signaling—that could be especially consequential in stromal-rich organs such as the liver [3,4].

Liver architecture imposes spatial constraints—periportal/pericentral zonation, sinusoidal flow, and perisinusoidal niches—that modulate how secreted regulators are produced, diffuse, and are cleared, making binding proteins logical determinants of effective signal range [5]. Technological advances now allow these questions to be tackled directly: single-cell atlases define lineage-specific expression programs, while spatially resolved transcriptomics preserves tissue coordinates to relate ligand/binding-protein localization to vascular landmarks and metabolic zones [6,7,8]. In parallel, tumor-stroma research has reframed fibroblasts as heterogeneous, state-dependent regulators of growth-factor availability and therapeutic response—an insight with clear implications for hepatic stellate cells and cancer-associated fibroblasts in liver disease [9,10].

Among IGFBPs, IGFBP-6 is distinguished by high affinity for IGF-II and a repertoire of IGF-independent actions; however, its liver-specific compartmentalization, spatial gradients, and stimulus-dependent regulation remain incompletely mapped across homeostasis, injury, and malignancy [4].

This review synthesizes emerging multi-omic and spatial evidence to delineate IGFBP-6 cell-of-origin, zonation, and regulation in the liver; integrates these data with stromal biology to propose mechanisms for disease-stage-specific roles; and outlines translational opportunities—from biomarker strategies to rational patient selection—in primary liver cancers. To aid future study design, we also highlight methodological considerations—combining spatial transcriptomics with vascular landmarking, post-translational-modification-aware proteomics, and quantitative systems frameworks—to resolve IGFBP-6 function across physiological gradients and perturbed microenvironments.

## 2. The IGF Signaling Axis and IGFBP-6

### 2.1. Overview of the IGF-II Receptor Landscape

The insulin-like growth factor (IGF) system is a conserved, multifunctional network governing cellular growth, survival, differentiation, and metabolism. It comprises the ligands IGF-I and IGF-II; the signaling receptors IGF-1R and insulin receptor (IR) isoforms; the IGF-II receptor IGF2R (also termed the cation-independent mannose-6-phosphate receptor, CI-MPR/CD222); and six high-affinity IGF-binding proteins (IGFBPs) that regulate ligand bioavailability and receptor activation [1]. IGF-II is a potent mitogen with essential roles in fetal development; in adult tissues, its expression is typically restrained but is frequently reactivated in cancer, where it can sustain signaling through IGF-1R and the IR isoform A (IR-A) to support proliferation and survival [11,12]. Beyond promoting oncogenic growth, IGF-II can remodel the tumor microenvironment and contribute to immune evasion, including fibroblast-dependent mechanisms that blunt responses to immunotherapy [13]. In parallel, IGF2R binds IGF-II with high affinity but lacks intrinsic tyrosine-kinase activity and is widely viewed as a major regulator of IGF-II bioavailability through ligand binding, internalization, and trafficking (in addition to its canonical role in mannose-6-phosphate-dependent lysosomal enzyme sorting) [14,15,16].

IGF-1R is a transmembrane tyrosine kinase of the insulin receptor family; ligand binding induces receptor autophosphorylation, adaptor recruitment (IRS, Shc), and activation of PI3K–AKT and MAPK/ERK cascades that mediate mitogenic, anti-apoptotic, and metabolic outputs [17,18,19]. While IGF-1R is most potently activated by IGF-I, IGF-II can also engage IGF-1R and IR-A to elicit signaling, as supported by structural and mutational analyses [20,21]. Differential engagement of IGF-1R and IR-A helps diversify downstream programs and underpins IGF-II-driven proliferation, survival, and microenvironmental interactions [21]. In disease, dysregulated IGF-II/IGF-1R signaling contributes to tumorigenesis, metastasis, and therapy resistance via autocrine or paracrine IGF-II that sustains receptor activation, angiogenesis, and apoptosis evasion [19]. Importantly, IGF2R can modulate the effective “free” IGF-II available to signal receptors by buffering and clearing ligand; thus, changes in IGF-II binding proteins (including IGFBP-6) may influence not only IGF-1R/IR-A signaling but also IGF-II trafficking/clearance dynamics [14,15]. IGF-II also participates in non-canonical processes such as maintenance of cancer stem-like states via the IGF-II/IGF-1R/Nanog axis [20].

### 2.2. IGFBP-6: Dual IGF-Dependent and IGF-Independent Actions

IGFBPs modulate IGF signaling by shaping ligand distribution, half-life, and receptor accessibility. Among them, IGFBP-6 is distinctive for its very high affinity for IGF-II and operates through two broad modes: (i) IGF-dependent sequestration, limiting IGF-II access to receptors; and (ii) IGF-independent signaling, involving alternative partners and pathways [22]. Because IGF2R/CI-MPR is a major IGF-II-binding/trafficking receptor, IGFBP-6-dependent changes in “free” IGF-II are also expected to intersect with IGF2R-mediated ligand handling, although this relationship has not been systematically examined in liver contexts [14,15]. In its IGF-dependent role, IGFBP-6 suppresses IGF-II-driven proliferation, survival, migration, and differentiation by reducing ligand engagement of IGF-1R (and IR-A), with consequent attenuation of downstream PI3K–AKT and RAS–MAPK signaling; in tumor models (e.g., glioma), enforced IGFBP-6 reduces IGF-1R/AKT activation and tumor cell expansion [23,24,25].

IGF-independent activities reflect additional molecular interactions. IGFBP-6 can bind cell-surface proteins such as prohibitin-2, translocate to the nucleus, and influence non-IGF pathways—including Hedgehog and Wnt—affecting migration, angiogenesis, apoptosis, and immune modulation without requiring IGF-II binding [26,27,28,29]. In some mesenchymal and rhabdomyosarcoma contexts, IGFBP-6 can activate MAPK/ERK and promote cell migration rather than proliferation, emphasizing context dependence [27,30]. Crosstalk with TGF-β adds further nuance: TGF-β downregulates IGFBP-6 in osteoblasts, whereas in fibrotic or inflammatory milieus, cytokine-driven IGFBP-6 can contribute to fibroblast activation and extracellular-matrix remodeling [28,29,31].

Interactions with Wnt/β-catenin are described primarily in oncogenic settings: IGFBP-6 overexpression can dampen β-catenin-dependent transcription and proliferation, while its loss may enhance Wnt activity and clonogenic growth, potentially via upstream kinase signaling [28,32]. In addition to functional crosstalk, there is primary evidence that canonical Wnt/β-catenin signaling can directly regulate IGFBP-6 transcription in fibroblast-like tumor settings. In desmoid tumors (aggressive fibromatosis), stabilized β-catenin represses IGFBP-6 expression, and promoter/EMSA studies identified functional β-catenin/T-cell factor (TCF)-responsive elements in the human IGFBP-6 promoter, supporting direct transcriptional repression by the β-catenin/TCF complex [33]. These findings provide a mechanistic precedent for Wnt-pathway control of IGFBP-6, but the direction and cell-type specificity of this regulation cannot be assumed to generalize to liver lineages without direct testing.

Hypoxic regulation further integrates IGFBP-6 into stress-response networks: hypoxia-responsive elements within the IGFBP6 promoter mediate hypoxia inducible factor-1α (HIF-1α)-dependent induction in endothelial cells, where IGFBP-6 exhibits anti-angiogenic effects and contributes to adaptation to oxygen deprivation [28,34].

In sum, IGFBP-6 is a context-specific regulator that can either buffer IGF-II to blunt IGF-1R output or engage IGF-independent programs that rewire motility, angiogenesis, apoptosis, and immune tone. Through intersection with IGF-1R, MAPK, TGF-β, Wnt/β-catenin, and hypoxia/HIF pathways, IGFBP-6 sits at a regulatory crossroads linking growth control, stress adaptation, and microenvironmental remodeling.

## 3. IGFBP-6 in the Liver: Cellular Compartmentalization and Regulation

### 3.1. Cellular Sources Within the Hepatic Microenvironment

**Figure 1:**
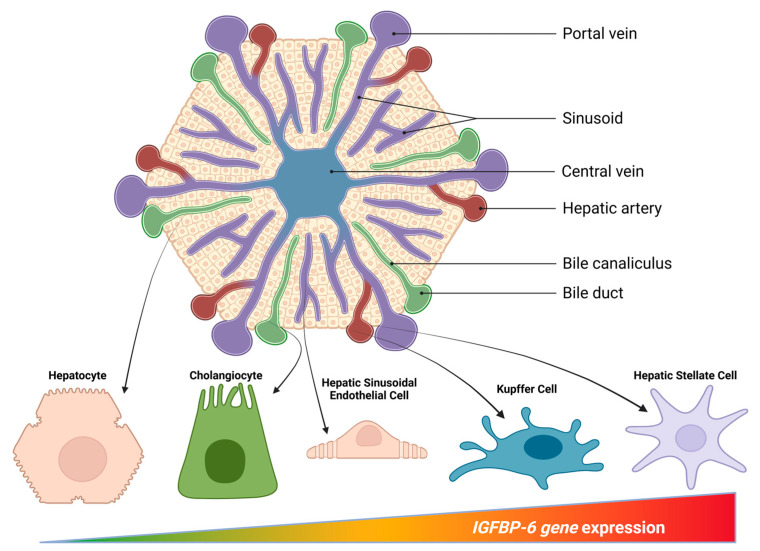
Hepatic lobule schematic highlighting IGFBP-6 expression across cell types and the portal–central axis. Central vein-centered lobule with portal triads shows portal vein and sinusoids (purple), hepatic artery branches (red), central vein (blue), and bile ducts/canaliculi (green). Representative cell cartoons indicate the main hepatic lineages—hepatocyte, cholangiocyte, liver sinusoidal endothelial cell (LSEC), Kupffer cell, and hepatic stellate cell (HSC). The color bar denotes a qualitative IGFBP-6 gene-expression gradient, emphasizing stromal-skewed enrichment (highest in HSCs, lower in Kupffer cells/LSECs/cholangiocytes, lowest in hepatocytes) and a perisinusoidal spatial context along the portal–central axis. Created in Biorender. Coda, A.R.D. (2025) https://app.biorender.com/illustrations/6914ba24a9ea3d0e174ddfaf, accessed on 27 December 2025.

IGFBP-6 is present in the liver across multiple parenchymal and non-parenchymal compartments, with transcript detection in hepatocytes, Kupffer/macrophage fractions, endothelial cells, cholangiocytes, fibroblasts and immune cells in single-cell reference atlases [6,35] (Figure 1).

The overall pattern is multicellular but stromal-skewed, with fibroblast/stellate and smooth-muscle-like clusters consistently registering *IGFBP-6* reads alongside lower signals in additional lineages [36]. Although *IGFBP-6* is not emphasized in the original atlas publications, inspection of the publicly available single-cell liver expression matrices indicates stromal-skewed *IGFBP-6* transcripts, with the highest signal in stellate/mesenchymal compartments and lower levels in additional lineages [6,35].

Multiple orthogonal datasets converge on hepatic stellate cells (HSCs)—particularly activated HSCs (aHSCs)/myofibroblast-like states that emerge during fibrogenesis—as the dominant intrahepatic compartment with enriched *IGFBP-6* transcripts [31]. In mouse and human systems, primary and culture activation of primary HSCs is accompanied by increased *Igfbp6/IGFBP-6* expression relative to hepatocytes or Kupffer cells, and several fibrosis datasets report higher *IGFBP-6* signal in contexts characterized by stellate activation (e.g., toxin- or virus-associated injury models) [31]. Earlier primary-cell work showed that HSCs elaborate IGF-axis components and IGF-binding proteins, establishing the stellate lineage as a physiological source of IGFBPs in liver [37]. Where the literature does not explicitly separate quiescent HSCs (qHSCs) from aHSCs, we refer to “HSCs” and note that the inferred state depends on the experimental context (homeostatic vs. fibrotic/activated).

Beyond stellate cells, cholangiocytes and liver sinusoidal endothelial cells (LSECs) display detectable *IGFBP-6* mRNA in single-cell liver resources, indicating that biliary and vascular niches also contribute to the hepatic IGFBP-6 pool [36]. Single-cell atlases resolving Kupffer cells, monocyte-derived macrophages and lymphoid subsets in healthy human liver enable attribution of low-level immune-compartment *IGFBP-6* transcript signals and provide baselines for inflammatory re-programming in disease [35]. Methodologically oriented surveys of human liver scRNA-seq further support a distributed expression pattern, while underscoring that cell-state and platform differences can modulate detection sensitivity for moderately expressed genes like *IGFBP-6* [38]. We therefore specify throughout whether we refer to transcript-level enrichment (scRNA/spatial) versus protein-level measurements.

In desmoplastic primary liver cancers, especially cholangiocarcinoma, scRNA-seq analyses identify cancer-associated fibroblast (CAF) subsets in which *IGFBP-6* transcripts are enriched within broader co-expression programs (“modules”) that include IGF/IGFBP-axis genes together with inflammatory and matrix-remodeling markers [39]. Here, “module” refers to a co-expressed gene set inferred from the single-cell dataset; the presence of IGFBP-6 within such a program indicates stromal association but does not establish IGFBP-6 as a functional driver. Accordingly, these data place IGFBP-6 within CAF/stromal transcriptional programs and are consistent with (but do not prove) a paracrine contribution to the tumor microenvironment [39]. Broader tumor-stromal studies show that CAFs secrete IGF/IGFBP ligands that rewire signaling and drug response in adjacent cancer cells, supporting a mechanistic niche for stromal IGFBP-6 in liver tumor microenvironments [40]. Cholangiocarcinoma (CCA) microenvironment reviews compiling single-cell and bulk data likewise list *IGFBP-6* among fibroblast-enriched transcripts in CAF subsets linked to inflammation and matrix remodeling [41].

At the protein level, IGFBP-6 is a secreted factor with predominant extracellular localization, potentially reflecting intracellular and/or nuclear pools of IGFBP-6, consistent with the presence of a nuclear localization signal and reports of context-dependent nuclear localizations [42]. We note, however, that the cited study does not demonstrate distinct IGFBP-6 isoforms; rather, it supports subcellular compartmentalization as a hypothesis requiring direct confirmation in liver-relevant model. General IGFBP biology places these proteins in the extracellular milieu where they modulate IGF bioavailability, and IGFBP-6 conforms to this secretory paradigm while showing a strong preference for IGF-II [23]. Emerging work indicates that IGFBP-6 can also be released in extracellular vesicles under stress conditions, adding another route for intercellular transport that could operate in inflamed or fibrotic liver [43].

Circulating measurements capture disease-linked changes in IGFBP-6 abundance, but tissue-resolved data indicate that shifts in stromal composition—particularly HSC/CAF expansion—likely dominate hepatic contributions to the blood pool [31]. Given this multicompartment distribution, assigning functional roles to IGFBP-6 in the liver requires integration of single-cell atlases with perturbational models that track stellate activation and stromal remodeling across the porto-central axis.

### 3.2. Spatial Context and Lobular Zonation

The liver lobule is spatially organized along the portal–central axis, with metabolic and signaling programs partitioned into periportal and pericentral zones that shape how any secreted regulator—including IGFBP-6—is produced, encounters targets, and is cleared [44,45]. Direct experimental data on lobule-resolved IGFBP-6 protein distribution and function remain limited; therefore, this section is presented as a working framework that integrates established principles of liver zonation with atlas-based indications of IGFBP6 expression to generate testable hypotheses. Where statements go beyond direct IGFBP-6 evidence, we explicitly flag them as inferences or predictions.

**Figure 2:**
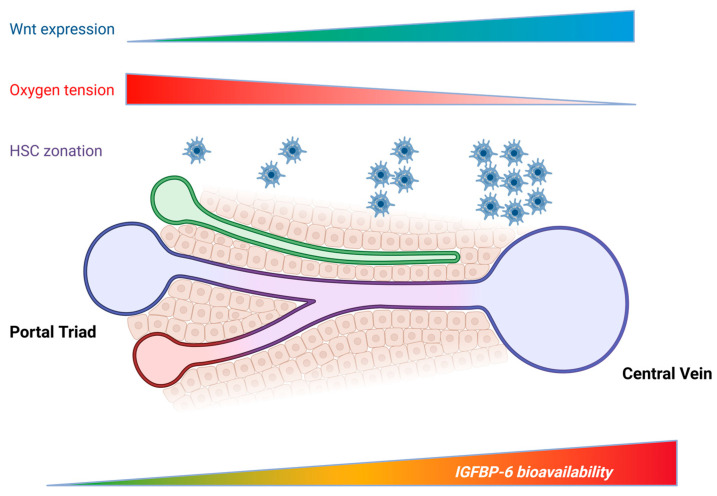
Hypothesis-driven spatial model of IGFBP-6 bioavailability along the portal–central axis. Schematic sinusoid from portal triad (left) to central vein (right) overlaid with representative lobular gradients (e.g., Wnt activity rising pericentrally and oxygen tension falling toward the central vein). The purple band denotes the perisinusoidal space containing hepatic stellate cells (HSCs), which are distributed along the sinusoid in homeostasis but comprise heterogeneous sub-states whose activation and local expansion can become spatially biased depending on microanatomical niche and disease etiology (periportal vs. pericentral injury patterns). The bottom bar illustrates a conceptual, not experimentally established possibility that local IGFBP-6 availability may vary across lobular space, motivated by (i) reported enrichment of IGFBP-6 transcripts in mesenchymal/stromal compartments in single-cell datasets and (ii) the established role of HSCs in shaping lobular signaling landscapes. This schematic is not intended to imply a fixed baseline gradient of HSC abundance, nor a validated monotonic periportal-to-pericentral IGFBP-6 gradient. Created in Biorender. Coda, A.R.D. (2025) https://app.biorender.com/illustrations/6917182b716812d970ff5e89, accessed on 27 December 2025.

Core patterning depends on Wnt/β-catenin activity that is highest pericentrally and suppressed periportally, establishing zone-specific transcriptional identities and metabolic specializations [46]. Because Wnt/β-catenin activity is intrinsically zonated (highest pericentrally), it provides a plausible upstream mechanism that could contribute to spatial patterning of Wnt-responsive genes—including, potentially, *IGFBP-6*. Notably, in fibroblast-derived desmoid tumor cells, stabilized β-catenin/TCF represses *IGFBP-6* via TCF-responsive promoter elements [33]. If a similar regulatory logic operated in specific liver-resident cell states, one could predict zone-biased *IGFBP-6* transcription (direction depending on whether β-catenin acts as a repressor or activator in the relevant hepatic lineage). However, liver non-parenchymal zonation is multi-factorial and state-dependent; therefore, any Wnt-linked *IGFBP-6* zonation should be treated as hypothesis-generating until validated using spatial transcriptomics plus in situ protein detection and Wnt-pathway perturbation. An opposing oxygen gradient from portal (high O_2_) to central (low O_2_) veins modulates hypoxia-inducible factor signaling and intersects with Wnt and Hedgehog pathways, further reinforcing zonation at the level of both hepatocytes and stromal cues (Figure 2) [47,48].

However, non-parenchymal zonation—particularly HSC heterogeneity—appears multi-dimensional and is unlikely to be explained by oxygen availability alone. Increasing evidence supports distinct HSC sub-states distributed along the porto-central axis and across microanatomical niches (perisinusoidal vs. peribiliary/perivascular positions), shaped by combinations of morphogen signaling, ECM composition, vascular proximity, inflammatory inputs, and injury context. Accordingly, any spatial model for IGFBP-6 should be considered hypothesis-generating until directly validated at the protein level.

Recent mechanistic work demonstrates that HSCs actively pattern zonation by supplying R-spondin-3 (RSPO3) to boost pericentral Wnt signaling, thereby controlling hepatocyte gene expression, lobular architecture, liver size, and detoxification capacity [49]. Selective deletion of Rspo3 in HSCs phenocopies HSC depletion, disrupting zonation programs and worsening alcohol-associated and metabolic steatosis, positioning perisinusoidal HSCs as organizers of the spatial transcriptome [49]. In parallel, several single-cell liver datasets suggest that *IGFBP-6* mRNA is enriched in stromal/mesenchymal cell states (including HSC-like and fibroblast-like populations [31]. Taken together, these observations motivate—but do not prove—the hypothesis that IGFBP-6 bioavailability could vary across lobular spaces in ways influenced by perisinusoidal stromal geography and RSPO-Wnt patterning. Importantly, mRNA enrichment does not necessarily predict protein abundance, secretion, extracellular binding, or local activity, and these relationships will require direct validation.

Spatial omics now maps zone-resolved expression at near-cellular resolution, revealing conserved and species-specific periportal/pericentral gene sets that provide a scaffold for interrogating secreted modulators like IGFBP-6 [50]. Emerging spatial atlases from healthy live donors extend these maps and show fine-grained zonation of non-parenchymal cells, enabling the localization of stromal signals and their proximity to vascular landmarks relevant for gradient-driven regulation [51]. Dedicated public resources (e.g., LISTA) integrate spatial and single-cell layers across homeostasis and regeneration, offering queryable contexts to assess whether *IGFBP-6* transcripts show reproducible spatial patterns as datasets accumulate [52].

Zonation is not static: inflammatory and fibrotic remodeling distort the porto-central template, with pericentral Wnt programs, oxygen tension, and sinusoidal architecture all shifting as disease progresses [44,48]. In fibrotic states, expansion and activation of perisinusoidal HSCs/CAF-like cells change the spatial distribution of stromal secretomes; we therefore hypothesize that local *IGFBP-6* pools (tissue vs. circulating; free vs. matrix-associated) may be reshaped during fibrosis, although direct spatial protein data are currently lacking (inference) [49]. Single-cell and fixed-cell atlases of fibrosis progression further show that spatially restricted mesenchymal programs intensify with stage, underscoring the need to interpret any *IGFBP-6* “up/down” signal through a zonation lens rather than bulk averages alone [53]. However, whether IGFBP-6 protein itself exhibits zonated extracellular deposition, altered clearance, or disease/stage-dependent redistribution remains unknown.

Methodologically, testing IGFBP-6 zonation will require combining spatial transcriptomics with in situ protein measurements and vascular landmarking, then overlaying Wnt and hypoxia reporters to align IGFBP6 with periportal/pericentral identities [50]. Because HSC-secreted RSPO3 sculpts hepatocyte zones, perturbing RSPO-Wnt (genetically or pharmacologically) provides a causal framework to ask whether IGFBP-6 transcription, IGFBP-6 protein localization, or IGF-II binding capacity shifts with zone re-patterning [49]. Finally, single-cell liver resources that already catalog IGFBP6 across non-parenchymal and biliary lineages offer immediate priors for selecting regions of interest and powering spatial studies that explicitly test periportal vs. pericentral enrichment.

### 3.3. Regulation by Profibrotic, Metabolic, and Inflammatory Cues

#### 3.3.1. TGF-β and Growth-Factor Programs in Stellate Cells

Transforming growth factor-β (TGF-β) is the canonical driver of HSC activation, and recent mechanistic work identifies IGFBP-6 as a fibrosis-specific effector upregulated in activated HSCs and functionally linked to the TGF-β/Suppressor of mothers against decapentaplegic homolog (SMAD) pathway in mouse models and human tissue [31]. In that study, pharmacologic inhibition of IGFBP-6 attenuated SMAD signaling and extracellular matrix deposition in vivo, placing IGFBP-6 downstream of profibrotic cues and upstream of matrix remodeling [31].

Earlier primary HSC work established that activated stellate cells constitutively produce IGF-binding proteins and are responsive to TGF-β family signals, providing historical context for IGFBP-6 being embedded in growth-factor-regulated HSC programs [37]. More broadly, the stellate activation literature positions TGF-β and platelet-derived growth factor (PDGF) as central inputs that remodel HSC transcriptional states and secretomes, consistent with IGFBP-6 induction during fibrogenesis [54].

Reviews focused on IGFBP-6 across tissue repair and fibrosis also highlight bidirectional relationships with TGF-β signaling in stromal cells, reinforcing a model in which profibrotic cytokines transcriptionally and functionally tune IGFBP-6 [29].

#### 3.3.2. Inflammatory Cytokines and Innate Stimuli

Pro-inflammatory cytokines can modulate IGFBP expression; notably, interleukin-1β (IL-1β) and tumor necrosis factor (TNF) increased IGFBP-6 production in cultured human fibroblasts, while reducing IGFBP-3 and IGFBP-4 [29]. Whether analogous regulation occurs in liver-resident mesenchymal cells (e.g., HSCs/CAFs) remains to be directly tested [55]. Functionally, IGFBP-6 modulates cytokine programs in epithelial and immune settings, with neutralization amplifying lipopolysaccharide (LPS)-triggered inflammatory gene expression—an effect that supports a feedback role for IGFBP-6 in restraining excessive inflammation [56,57]. Stromal studies further show that IGFBP-6 can regulate α-smooth muscle actin (α-SMA), TGF-β, and fibroblast activation protein expression in mesenchymal cells exposed to innate and morphogen signals, connecting inflammatory and profibrotic axes at the level of the fibroblast/CAF phenotype [58].

#### 3.3.3. Hypoxia, Redox Status, and HIF Signaling

Peri-central hypoxia is a defining feature of the lobule, and hypoxia upregulates *IGFBP-6* through the HIF-1 in human vascular endothelium, establishing a direct mechanistic link between oxygen tension and *IGFBP-6* transcription that is likely to operate in the hepatic microvasculature [34]. Additional hypoxia studies show IGFBP6 responsiveness to low oxygen and endoplasmic reticulum (ER)-stress signaling in other cell types, supporting a conserved hypoxia/inositol-requiring enzyme 1 (IRE1) axis that could intersect with liver injury where ER stress and hypoxia co-occur [59].

A recent synthesis places IGFBP-6 within redox-sensitive regulatory networks tied to immune activation and fibrosis, aligning with hypoxia-driven shifts expected in steatotic and fibrotic livers [60].

#### 3.3.4. Metabolic Cues and Nutrient Sensors

Metabolic remodeling during steatosis and lipotoxic stress is accompanied by lactate accumulation and altered substrate fluxes, and IGFBP-6 is responsive to metabolic signals such as lactate in immune cells, where it shapes transporter expression and inflammatory tone—suggesting a route by which hepatocellular and stromal metabolism could tune IGFBP-6 in the liver [61]. IGF-axis cross-talk with metabolic pathways in HSCs is well described, and IGFBP-family expression (notably IGFBP-3) changes during early stellate activation [62]. Together with evidence that *IGFBP-6* is enriched in activated HSC/mesenchymal compartments in liver datasets, these observations motivate testing whether metabolic rewiring also regulates IGFBP-6 during fibrogenesis [62]. Comprehensive hepatology reviews of epigenetic regulation in fibrosis further argue that nutrient-linked epigenetic writers and erasers modulate fibrogenic genes, a framework that can encompass IGFBP6 regulation via chromatin remodeling during disease progression [63].

#### 3.3.5. Post-Translational Modification and IGF-II Sequestration

Viral hepatitis provides a clear example of post-translational control: O-β-GlcNAc modification of IGFBP-6 at Ser204 reduces IGF-II binding, implying that inflammatory and metabolic fluxes through the hexosamine pathway can shift IGFBP-6’s capacity to sequester IGF-II in infected livers [64]. These post-translational changes are proposed to increase free IGF-II bioavailability and thereby favor pro-growth signals implicated in hepatocarcinogenesis, linking innate/infectious inflammation to growth-factor reprogramming through IGFBP-6.

Biochemical overviews underscore that IGFBP-6 is uniquely IGF-II-preferring among IGFBPs, so any post-translational modification that weakens IGF-II affinity would be expected to have disproportionate consequences for IGF signaling in liver disease [65].

#### 3.3.6. Integration Across Pathways and Disease Stages

Taken together, these data support a model in which TGF-β signaling, inflammatory cytokines, hypoxia/HIF activity, and metabolic stress converge to induce IGFBP-6 in HSCs and other hepatic compartments, with downstream effects that combine IGF-dependent sequestration and IGF-independent stromal modulation (Table 1) [29,31,34,60].

Because both transcriptional control and post-translational modification influence IGFBP-6 abundance and binding properties, compartment-aware, stimulus-resolved assays are needed to interpret circulating and tissue IGFBP-6 during steatosis, fibrosis, and viral hepatitis.

## 4. IGFBP-6 in Liver Diseases

Non-neoplastic liver diseases remodel endocrine, metabolic, and stromal circuits in ways that plausibly alter IGFBP-6 production, processing, and release into blood, but direct evidence is uneven across etiologies and often stage-dependent, necessitating disease-specific appraisal and careful interpretation of serum versus intrahepatic signals (Table 2).

### 4.1. Metabolic Dysfunction-Associated Steatotic Liver Disease (MASLD)

MASLD (formerly non-alcoholic fatty liver disease, NAFLD) encompasses hepatic steatosis linked to metabolic dysfunction, and its inflammatory, progressive form is metabolic dysfunction-associated steatohepatitis (MASH); the nomenclature and diagnostic framework were established by a 2023 international Delphi consensus and incorporated into 2024 multisociety guidance [71,72]. Disease arises from nutrient surplus, insulin resistance, lipotoxicity, and immune–stromal activation culminating in steatohepatitis and fibrosis, with ongoing trials showing that weight loss and incretin-based therapies can modify histologic trajectories [73].

In a well-phenotyped human cohort, IGFBP-family profiling linked several IGFBPs to histologic severity; IGFBP-6 showed disease-related associations, supporting its inclusion in MASLD staging panels, albeit with divergent directions across proteins [66]. In HIV-associated MASLD, the growth hormone (GH)-releasing hormone analog tesamorelin reduced liver fat and prevented fibrosis progression while down-modulating hepatic gene sets involved in inflammation and tissue repair, providing mechanistic plausibility that IGF-axis modulators can shift the milieu in which IGFBP-6 operates [74,75]. Independent obesity-intervention literature confirms that circulating hepatokines/adipokines change with fat loss and metabolic improvement, reinforcing the expectation that IGFBP-6 behaves as a state-dependent signal rather than a static trait marker in metabolic liver disease [76].

### 4.2. Alcohol-Associated Liver Disease (ALD)

ALD spans simple steatosis, steatohepatitis, fibrosis, and cirrhosis driven by sustained alcohol exposure; contemporary guidance recommends “alcohol-associated” terminology and outlines graded management across the spectrum [77]. Injury reflects ethanol and acetaldehyde toxicity, gut–liver axis changes, oxidative stress, and stellate-cell activation, with acute alcoholic hepatitis representing a high-mortality inflammatory peak [78].

Classic work in alcoholic cirrhosis documented broad perturbations of the IGF/IGFBP system in serum, indicating endocrine remodeling of the axis in advanced ALD, although IGFBP-6-specific measurements were limited or absent in early series [79,80]. More recent ALD pathobiology underscores HSC activation as a central driver of fibrogenesis, indirectly strengthening the rationale to quantify IGFBP-6—given its stellate-enriched expression—in stage-resolved ALD cohorts, even though definitive human data are still sparse [81].

### 4.3. Viral Hepatitis

Chronic hepatitis B virus (HBV) and hepatitis C virus (HCV) infections cause progressive necroinflammation and fibrosis, remain major global causes of cirrhosis, and remodel growth-factor networks including the IGF axis [82]. Persistent viral replication and immune activation drive hepatocellular injury, with fibrosis risk set by host, viral, and environmental factors despite the availability of curative HCV therapies and effective HBV suppression [82].

A case–control study in chronic HCV that quantified all seven IGFBPs found group-level differences concentrated in other IGFBPs, but IGFBP-6 varied with fibrosis stage (higher at advanced F3/F4 versus controls), implying non-linear behavior across disease progression [67]. Mechanistically, IGFBP-6 can be O-β-GlcNAc-modified at Ser204 during HBV/HCV infection, diminishing IGF-II binding and potentially increasing free IGF-II, which contextualizes how inflammatory/metabolic fluxes may tune IGFBP-6 function in chronic viral hepatitis [64].

### 4.4. Cholestatic and Autoimmune Biliary Disease

Primary biliary cholangitis (PBC) is a chronic autoimmune cholestatic disease marked by small-duct injury, cholestasis, and—without treatment—progression to biliary cirrhosis; European guidelines outline diagnostic criteria and first-line therapy [83]. Primary sclerosing cholangitis (PSC) is a fibro-inflammatory cholangiopathy with multifocal biliary strictures, strong association with inflammatory bowel diseases, and high risk of progressive cholestasis and cholangiocarcinoma; contemporary guidance emphasizes surveillance and complication management [84]. Both conditions feature immune-mediated bile-duct injury, cholestasis, and portal fibro-inflammation, with microbiome and barrier alterations highlighted in PSC [84].

Proteomic screens of PSC bile and serum reveal extensive remodeling but have not identified IGFBP-6 among top discriminatory markers to date, suggesting either subtle effects, compartmentalization below current detection thresholds, or cohort/platform variability [68]. PBC state-of-the-art reviews similarly prioritize immune-cholestatic signatures over IGFBP-6, indicating a present evidence gap that warrants targeted assays in biliary disease biobanks [85].

### 4.5. Autoimmune Hepatitis

Autoimmune hepatitis (AIH) is an immune-mediated inflammatory liver disease that affects all ages and can present from asymptomatic elevation of aminotransferases to acute liver failure; diagnosis integrates serology, histology, and exclusion of other causes, with corticosteroids as effective first-line therapy [85]. Loss of tolerance to hepatocellular antigens leads to T-cell-mediated injury and interface hepatitis, with genetic and environmental contributors variably implicated [86].

Recent machine-learning proteomics developed diagnostic panels that distinguish AIH from healthy and MASLD comparators, but IGFBP-6 has not emerged as a consistent component, indicating that if present its contribution may be secondary to dominant immune pathways [69]. Broader autoimmune-disease proteomics highlight methodological readiness for systematic IGFBP-6 quantification in AIH, supporting prospective inclusion even as current reports are limited [87].

### 4.6. Cirrhosis, Portal Hypertension, and Systemic Decompensation

Cirrhosis represents advanced scarring with architectural distortion and complications driven by portal hypertension; pathophysiology reflects increased intrahepatic resistance, sinusoidal remodeling, and hyperdynamic circulation [88,89]. Endothelial dysfunction, stellate-cell contraction, extracellular matrix deposition, and angiogenesis converge to raise portal pressure, with hepatic venous-portal gradient (HVPG) serving as the invasive gold standard for grading risk and monitoring therapy [90,91].

In advanced chronic liver disease, circulating IGF-1 and IGFBP-3 consistently fall and correlate with hepatic dysfunction and outcomes, establishing a robust endocrine signature against which IGFBP-6 should be interpreted [92,93,94]). Liver-sinusoidal endothelial cell remodeling and collagen IV deposition have been causally linked to portal hypertension, providing a microenvironmental context where stromal IGFBP-family dynamics—including IGFBP-6—could change with vascular remodeling, though direct IGFBP-6 data in this setting remain to be generated [95].

### 4.7. Hepatic Ischemia–Reperfusion (I/R)

Liver I/R injury—which accompanies transplantation and inflow control during major hepatectomy—features an ischemic phase followed by inflammation-dominated reperfusion damage and remains a major determinant of early allograft dysfunction and postoperative outcomes [96,97]. Reperfusion biology integrates neutrophil recruitment, oxidative burst, endothelial activation, cell-death programs, and damage-associated molecular pattern (DAMP)-driven cytokine cascades, providing multiple regulatory nodes at which secreted binding proteins could tune growth-factor availability [98].

Clinical and experimental observations already show IGF-axis remodeling during hepatic I/R: IGFBP-3 rises with injury severity and aggravates post-ischemic damage when overexpressed, while IGF-1 falls inversely with aminotransferase release, indicating that IGF/IGFBP dynamics are tightly coupled to tissue damage [99]. Although IGFBP-6 has not yet been systematically quantified in hepatic I/R cohorts, contemporary mechanistic reviews place IGFBP-6 within redox-sensitive immune programs—including links to oxidative stress responses and neutrophil activation—that are cardinal features of reperfusion pathology, justifying targeted measurement in this setting [60].

Family-level precedent further supports inducibility in hypoxia–stress milieus: IGFBPs exhibit hypoxia-responsive regulation in hepatocyte models, and best-practice I/R overviews identify HIF- and cytokine-driven transcription as key upstream layers to probe for IGFBP-6 [96,100].

Practically, PTM-aware proteomics and extracellular vesicle (EV) pipelines recommended for liver injury research can be applied to IGFBP-6 to determine whether modified or EV-carried IGFBP-6 better correlates with reperfusion injury scores or early graft dysfunction than total soluble protein alone [97].

### 4.8. Liver Regeneration

Liver regeneration after partial hepatectomy (PHx) is a highly orchestrated process that restores mass and function through a priming phase followed by proliferation and redifferentiation, with canonical triggers mapped by time-course transcriptomics and classic immediate-early gene surveys [101,102,103]. Growth-factor and cytokine circuits—including IL-6/STAT3—govern the priming response and are necessary for efficient hepatocyte cell-cycle entry in severe resection models [104].

Within this framework, the IGF/IGFBP system participates in regeneration dynamics, as shown by immediate-early induction of hepatic IGFBP-1 after 70% PHx and its transcriptional dependence on IL-6/STAT3 signaling in vivo [105,106]. Loss-of-function evidence underscores axis relevance: IGFBP-1 knockout mice display abnormal post-PHx regeneration with necrosis and a blunted DNA synthetic response, establishing that IGF-ligand buffering by IGFBPs influences hepatic regrowth [107].

Human and animal studies consistently catalog regeneration-linked surges of IGF system components, supporting targeted evaluation of additional binding proteins beyond IGFBP-1 during the priming and proliferative windows [103]. Direct human or murine data dissecting IGFBP-6 kinetics during PHx-driven regeneration remain limited, but two lines of evidence motivate focused study: (i) IGFBP-6 is a secreted IGF-II-preferring binder with IGF-independent actions in repair settings, and (ii) it is embedded in redox- and immunity-linked programs that are activated during regenerative priming [60,70]. Because IL-6/STAT3 is a central priming driver and IGFBP family members respond to this axis in the regenerating liver, IGFBP-6 constitutes a mechanistically plausible, yet understudied, readout to incorporate into PHx time-courses alongside cytokine and growth-factor panels [104,106]. Methodologically, state-of-the-art regeneration studies now combine bulk/single-cell RNA-seq with targeted proteomics, offering a practical path to add PTM-aware IGFBP-6 measurements (e.g., glyco-enriched parallel reaction monitoring/single reaction monitoring) to resolve whether its abundance or modification state tracks priming and proliferative transitions [103].

## 5. IGFBP-6 and Liver Cancers

Public cancer atlases consolidate that IGFBP-6 is variably expressed across tumors with context-specific survival associations, but liver-specific prognostic signals remain unresolved at the population level, underscoring the need for liver hepatocellular carcinoma/intrahepatic cholangiocarcinoma (LIHC/iCCA)-focused analyses rather than pan-cancer extrapolation [108].

### 5.1. IGFBP-6 and Hepatocellular Carcinoma

Hepatocellular carcinoma (HCC) arises on a background of chronic injury and cirrhosis in most patients and features recurrent activation of the IGF axis, with elevated ligand signaling and IGF-1R pathway engagement repeatedly implicated in tumor initiation, maintenance, and therapeutic vulnerability [109]. Comprehensive and recent reviews converge on a model in which increased IGF-II and reduced functional IGF-I bioactivity couple to PI3K/AKT–MAPK cascades in HCC, reinforcing the conceptual importance of proteins that regulate ligand availability, such as the IGFBPs [110]. Within this framework, IGFBP-6 is distinctive because it preferentially binds IGF-II, positioning it to modulate a ligand that is frequently upregulated in virally driven and metabolic HCC contexts [109].

In one antibody-array study, circulating IGFBP-6 showed only a modest decrease in patients with HCC (mean ~15%) than in those with chronic hepatitis, and this signal was not validated by an orthogonal quantitative assay [111]. Accordingly, these data are insufficient to infer broad IGF-axis remodeling or altered IGF-II bioavailability, but they do motivate more rigorous, assay-validated studies to determine whether IGFBP-6 changes reproducibly in defined HCC contexts. Mechanistically, hepatitis B and C infections introduce a biochemical lever on IGFBP-6 function: O-β-GlcNAc modification at Ser204 weakens IGF-II binding, which is predicted to increase free IGF-II and thereby potentiate IGF-dependent oncogenic signaling in infected livers [64]. This post-translational change dovetails with experimental evidence that IGF signaling fosters stem-like phenotypes and self-renewal in HCC models, indicating a plausible route by which infection-linked glycosylation of IGFBP-6 could feed forward into tumor aggressiveness [112].

Collectively, these data support a dual perspective: IGFBP-6 participates in HCC biology both as a candidate circulating analyte whose levels diverge from chronic hepatitis and as a biochemical gatekeeper whose O-GlcNAc status may tune the fraction of bioactive IGF-II available to tumor and stromal receptors.

### 5.2. IGFBP-6 and Intrahepatic Cholangiocarcinoma

Intrahepatic cholangiocarcinoma (iCCA) is an aggressively desmoplastic cancer in which CAFs dominate the tumor microenvironment (TME) and orchestrate matrix remodeling, soluble-factor gradients, and immune composition [39].

Single-cell atlases of human iCCA reveal intense stromal–epithelial cross-talk and substantial CAF heterogeneity, providing the cellular architecture in which IGF-axis modulators—including IGFBP family members—can shape ligand availability and signal routing to cholangiocarcinoma cells [113].

Across cancers, state-of-the-art single-cell and systems reviews underscore that CAFs are not monolithic but stratify into inflammatory, myofibroblastic, and “normal-like” states with distinct secretomes and receptor–ligand connectomes, implying state-dependent expression and action of IGFBPs in biliary tumors [114]. Functionally, CAFs can secrete IGF-binding proteins that alter signaling dependencies and drug responses in neighboring cancer cells, as demonstrated in controlled models where recombinant IGFBPs phenocopied CAF-conditioned effects—directly validating IGFBPs as paracrine effectors of the TME [40].

In cholangiocarcinoma specifically, focused reviews emphasize CAF dominance and growth-factor handling as druggable stromal functions, situating IGF/IGFBP biology—including IGFBP-6—within tractable paracrine networks that might be leveraged for therapy or biomarker development [39]. More recent single-cell landscapes that parse extrahepatic versus intrahepatic subtypes further document microenvironmental diversity, supporting spatially resolved assays to localize IGFBP-6 in CAF niches and along invasive fronts where growth-factor gradients and matrix alignment intersect [115].

### 5.3. Translational Implications: Biomarkers, Patient Selection, and PTM-Aware Targeting

Biomarker-wise, the observed decrease in serum IGFBP-6 in HCC relative to chronic hepatitis encourages evaluation of IGFBP-6 as an adjunct classifier, but studies must control for underlying etiology, fibrosis stage, and antiviral treatment to avoid confounding axis-level shifts [111]. Given evidence that O-β-GlcNAc at Ser204 reduces IGF-II binding by IGFBP-6, PTM-aware assays (e.g., glyco-specific enrichment coupled to targeted MS) are warranted to test whether modified IGFBP-6 better stratifies virally driven HCC or predicts IGF-pathway activity than total IGFBP-6 alone [64].

Therapeutically, repeated attempts to inhibit the IGF axis in HCC underscore that pathway blockade is feasible but demands precise patient selection and on-pathway pharmacodynamic readouts; integrating IGFBP-6 abundance and Ser204 O-GlcNAc status could refine these selection criteria by indexing how much IGF-II is effectively sequestered in vivo [110].

In iCCA and stroma-rich HCC, CAF-resolved spatial profiling of IGFBP-6 (RNA/protein) should be paired with CAF-state scoring and ligand–receptor inference to identify stromal neighborhoods where IGFBP-driven paracrine control of IGF signaling is most active and therapeutically exploitable [113,114]. Because CAFs can secrete IGFBPs that sensitize or desensitize tumors to targeted agents in a context-dependent fashion, co-targeting strategies that harness or mimic IGFBP-mediated modulation deserve investigation in liver cancers, especially where IGF-1R/FAK dependencies are evident [40].

## 6. Conclusions

IGFBP-6 is best viewed as a context-dependent regulator of liver biology, shaped by three coordinates: cell of origin (stellate/mesenchymal enrichment), place (periportal–pericentral zonation), and state (fibrosis, hypoxia/redox, inflammation, metabolism). In non-neoplastic disease, it behaves as a state sensor, so serum levels without spatial/tissue context are easy to misread and the timing of sampling matters. In cancer, IGFBP-6 functions both as a biochemical gatekeeper of IGF-II (via sequestration and PTMs that loosen the trap) and as a stromal organizer within CAF niches that modulate paracrine signaling. The field should now shift from measuring abundance to resolving function and location—using PTM-aware assays, EV profiling, and spatial readouts tied to IGF-II activity—with translational emphasis on composite biomarkers, pathway-aligned patient selection, and context-guided “tuning” of IGF-II availability rather than blunt pathway blockade. Looking ahead, priorities include longitudinal, zone-anchored human cohorts and causal perturbation models (RSPO–Wnt, oxygen/redox, immune–metabolic) to map when and where IGFBP-6 is determinant, plus preclinical testing of strategies that stabilize or disrupt IGFBP-6–IGF-II complexes under companion-diagnostic guidance.

## Figures and Tables

**Table 1:** Regulators of IGFBP-6: stimuli, context, and mechanism.

Stimulus/Context	Model/Cell Type	Direction on IGFBP-6	Mechanism/Pathway	Notes	References
TGF-β (fibrogenic cue)	Primary and cultured HSCs (mouse/human)	↑	SMAD2/3 signaling	Fibrosis-linked effector in HSCs	[31]
PDGF/profibrotic growth-factor milieu	HSC activation	↑ (inferred)	PDGFR–ERK/PI3K programs that co-activate HSC secretome	Supports inclusion of IGFBP-6 in growth-factor-responsive HSC programs	[54]
IL-1β, TNF (inflammatory cytokines)	Human stromal/epithelial systems	↑	NF-κB/inflammatory transcription	Non-hepatic primary data; plausible in liver injury	[29]
LPS/innate trigger with IGFBP-6 neutralization	Epithelial/immune models	Modulatory (loss → ↑ inflammatory genes)	Feedback control of cytokine programs	Suggests IGFBP-6 restrains excessive inflammation	[57]
Hypoxia	Human endothelium	↑	HIF-1-dependent transcription	Likely relevant to LSECs/pericentral zones	[34]
ER stress/IRE1 arm under hypoxia	Non-hepatic cells	↑	IRE1/XBP1-linked stress response	Converges with hypoxia in injured liver	[59]
Redox/oxidative stress linkage	Multi-tissue synthesis	Context-dependent	Redox-sensitive regulatory networks	Places IGFBP-6 within immune-fibrotic redox circuits	[60]
Lactate/metabolic reprogramming	Human immune cells	↑/reprograms function	Metabolic–transcriptional coupling	Route by which lipotoxic/steatotic liver may tune IGFBP-6	[61]
Early metabolic activation of HSCs	Cultured HSCs	Family shift (incl. IGFBPs)	Metabolic–ERK/AKT pathways	Supports nutrient/state sensitivity of IGFBP programs	[62]
O-GlcNAc at Ser204 (viral hepatitis)	HBV/HCV contexts; biochemical assays	↓ IGF-II affinity (functional)	Hexosamine pathway → O-GlcNAcylation	Alters sequestration without requiring transcriptional change	[64]

Abbreviations: TGF-β, transforming growth factor-β; HSC, hepatic stellate cell; SMAD, Suppressor of Mothers against decapentaplegic homolog; PDGF, platelet-derived growth factor; PDGFR, PDGF receptor; ERK, extracellular signal-regulated kinase; PI3K, phosphatidylinositol-3 kinase; IL-1β, interleukin-1β; TNF, tumor necrosis factor; NF-κB, nuclear factor-κB; LPS, lipopolysaccharide; HIF-1, hypoxia inducible factor-1; ER, endoplasmic reticulum; IRE1, inositol requiring enzyme 1; XBP1, X-box binding protein 1; AKT, protein kinase B; HBV, hepatitis B virus; HCV, hepatitis C virus.

**Table 2:** IGFBP-6 across non-neoplastic liver diseases.

Disease/Context	Core Pathogenesis	IGFBP-6 Signal	Stage Dependence	References
MASLD/MASH	Insulin resistance, lipotoxicity, immune–stromal activation → steatohepatitis/fibrosis	Cohort profiling shows associations of IGFBP-6 with histologic severity within the IGFBP family	Likely (signals track steatosis/inflammation severity)	[66]
ALD	Ethanol/acetaldehyde toxicity, gut–liver axis, ROS, HSC activation	No dedicated IGFBP-6 measurements in classic ALD studies (axis-level changes reported, IGFBP-6 usually absent)	Unknown	-
Chronic viral hepatitis	Persistent viral antigens and immune injury → fibrosis	In HCV, IGFBP-6 varies with fibrosis (↑ at F3–F4 vs. controls); in HBV/HCV, O-GlcNAc(Ser204) on IGFBP-6 reduces IGF-II binding	Yes (fibrosis-linked in HCV)	[67]
Cholestatic/autoimmune biliary disease	Immune-mediated bile-duct injury, cholestasis, portal fibro-inflammation	Large PSC proteomics: IGFBP-6 not among top discriminatory markers (signal likely subtle/compartmental)	Unclear	[68]
Autoimmune hepatitis	Loss of tolerance → T-cell-mediated interface hepatitis	IGFBP-6 not consistently included in recent ML proteomic panels	Unclear	[69]
Hepatic ischemia–reperfusion	Ischemia → reperfusion ROS, neutrophils, endothelium activation, DAMPs	IGFBP-6 not yet profiled in I/R cohorts; mechanistic review places IGFBP-6 in redox/immune programs relevant to I/R	Time-locked effects expected	[60]
Liver regeneration	IL-6/STAT3 priming → proliferation → redifferentiation	IGFBP-6 kinetics not defined; plausibility from IGFBP-6 repair biology	Time-dependent, but unknown	[60,70]

Abbreviations: MASLD, metabolic dysfunction-associated steatotic liver disease; MASH, metabolic dysfunction-associated steatohepatitis; ALD, alcohol-associated liver disease; ROS, reactive oxygen species; HSC, hepatic stellate cell; HCV, hepatitis C virus; HBV, hepatitis B virus; IGF, insulin growth factor; PSC, primary sclerosing cholangitis; ML, machine learning; DAMPs, danger-associated molecular patterns; I/R, ischemia–reperfusion; IL, interleukin; STAT3, signal transducer and activator of transcription 3.

## Data Availability

No new data were created or analyzed in this study.

## Abbreviations

The following abbreviations are used in this manuscript:

IGFBP-6	Insulin growth factor binding protein-6
IGF	Insulin growth factor
TGF-β	Tumor growth factor-β
PDGF	Platelet-derived growth factor
PTM	Post-translational modification
PI3K	Phosphatidylinositol 3-kinase
AKT	Protein kinase B
RAS	Rat sarcoma
MAPK	Mitogen-activated protein kinase
IGF-1R	IGF-1 receptor
CI-MPR	Cation-independent mannose-6-phosphate receptor
IR-A	Insulin receptor isoform A
TCF	T-cell factor
HIF-1α	Hypoxia inducible factor-1α
LSEC	Liver sinusoidal endothelial cell
HSC	Hepatic stellate cell
aHSC	Activated hepatic stellate cell
qHSC	Quiescent hepatic stellate cell
CAF	Cancer-associated fibroblast
CCA	Cholangiocarcinoma
RSPO3	R-spondin-3
SMAD	Suppressor of mothers against decapentaplegic homolog
IL-1β	Interleukin-1β
TNF	Tumor necrosis factor
LPS	Lipopolysaccharide
α-SMA	α-smooth muscle actin
ER	Endoplasmic reticulum
IRE1	Inositol requiring enzyme 1
PDGFR	PDGF receptor
ERK	Extracellular signal-regulated kinase
NF-κB	Nuclear factor-κB
HBV	Hepatitis B virus
HCV	Hepatitis C virus
MASLD	Metabolic dysfunction-associated steatotic liver disease
MASH	Metabolic dysfunction-associated steatohepatitis
ALD	Alcohol-associated liver disease
ROS	Reactive oxygen species
PSC	Primary sclerosing cholangitis
I/R	Ischemia–reperfusion
STAT3	Signal transducer and activator of transcription 3
GH	Growth hormone
PBC	Primary biliary cholangitis
AIH	Autoimmune hepatitis
HVPG	Hepatic venous-portal gradient
DAMP	Damage-associated molecular pattern
EV	Extracellular vesicle
PHx	Partial hepatectomy
LIHC	Liver hepatocellular carcinoma
iCCA	Intrahepatic cholangiocarcinoma
HCC	Hepatocellular carcinoma
TME	Tumor microenvironment
